# A Novel Peptide Driving Neurodegeneration Appears Exclusively Linked to the α7 Nicotinic Acetylcholine Receptor

**DOI:** 10.1007/s12035-024-04079-7

**Published:** 2024-03-14

**Authors:** Sanskar Ranglani, Sibah Hasan, Joanna Komorowska, Nathalia Mayag Medina, Kashif Mahfooz, Anna Ashton, Sara Garcia-Ratés, Susan Greenfield

**Affiliations:** Culham Science Centre, Neuro-Bio Ltd, Building F5, Abingdon, OX14 3DB UK

**Keywords:** α7 nAChR, T14, G Protein, Alzheimer’s Disease

## Abstract

T14, a 14mer peptide, is significantly increased in the pre-symptomatic Alzheimer’s disease brain, and growing evidence implies its pivotal role in neurodegeneration. Here, we explore the subsequent intracellular events following binding of T14 to its target α7 nicotinic acetylcholine receptor (nAChR). Specifically, we test how various experimental manipulations of PC12 cells impact T14-induced functional outcomes. Three preparations were compared: (i) undifferentiated vs. NGF-differentiated cells; (ii) cells transfected with an overexpression of the target α7 nAChR vs. wild type cells; (iii) cells transfected with a mutant α7 nAChR containing a mutation in the G protein-binding cluster, vs. cells transfected with an overexpression of the target α7 nAChR, in three functional assays – calcium influx, cell viability, and acetylcholinesterase release. NGF-differentiated PC12 cells were less sensitive than undifferentiated cells to the concentration-dependent T14 treatment, in all the functional assays performed. The overexpression of α7 nAChR in PC12 cells promoted enhanced calcium influx when compared with the wild type PC12 cells. The α7_345–348 A_ mutation effectively abolished the T14-triggered responses across all the readouts observed. The close relationship between T14 and the α7 nAChR was further evidenced in the more physiological preparation of ex vivo rat brain, where T30 increased α7 nAChR mRNA, and finally in human brain *post-mortem*, where levels of T14 and α7 nAChR exhibited a strong correlation, reflecting the progression of neurodegeneration. Taken together these data would make it hard to account for T14 binding to any other receptor, and thus interception at this binding site would make a very attractive and remarkably specific therapeutic strategy.

## Introduction

The α7 nicotinic acetylcholine receptor (α7 nAChR) is a subtype of nicotinic acetylcholine receptor that is abundant in the brain [1, 2]. It is involved in various physiological functions, including learning, memory, and synaptic plasticity and comprises of ligand-gated ion channels that are one of the most potent calcium ionophores in the brain [3]. In addition to their ionotropic action, α7 nAChRs can functionally couple to G proteins, enabling a downstream calcium signaling response that can persist beyond the expected time course of channel activation, triggering metabotropic-signaling responses [4–6].

Activation of the α7 nAChR has been shown to modulate cognitive function and memory processes [7]. A 14mer peptide, T14, derived from C-terminus of the enzyme acetylcholinesterase (AChE) has been implicated in the pathogenesis of Alzheimer’s disease (AD) via an allosteric site on the α7 nAChR [8]. T14 has a trophic role in early development via calcium signaling at the α7 nAChR; however, this process is proposed to turn excitotoxic when activated in the adult brain, given the significantly reduced tolerance to calcium influx in aged neurons [8–11]. Such an activation has been postulated to underlie the neurodegenerative process, specifically AD [12, 13]. NBP14, the cyclized version of T14 that blocks its linear counterpart by displacing it from the α7 nAChR, has shown therapeutical potential in the attenuation of cognitive impairment and amyloid production in 5xFAD mouse model of AD, further corroborating the implication of T14 in the pathogenesis of AD [13]. The C-terminus of AChE features various trypsin-like cleavage points, from which a 30mer peptide ‘T30’ can be cleaved and, within this sequence, a 14mer - T14 can be further derived by proteases [12, 14, 15]. T30 has shown identical bioactivity to T14 *in-vitro*, and ex vivo, and is used here due to better stability than T14 [14].

The original hypothesis that T14 may be acting via the α7 nAChR arose due to the unusual observation of the almost lockstep co-expression of the α7 nAChR and the parent molecule of T14 – AChE in postnatal rodent brain [16]. Since then, accumulating evidence has suggested that T14 and its parent T30 acts via the α7 nAChR [8–10, 17–19]. However, its dependence on the α7 nAChR mediated metabotropic responses remain unexplored. The alignment of the M3-M4 loop of the α7 nAChR protein sequence suggests that a G protein binding cluster (GPBC) is conserved in the α7 nAChR [6]. A dominant negative mutation in this GPBC, α7_345–348 A_, abolishes the receptor mediated activation of the G protein complex (consisting of Gα_q_ and Gβγ) in response to positive allosteric modulators (PAM) such as PNU120596 in PC12 cells [4, 5]. This configuration attenuates the ability of the receptor to trigger calcium induced calcium release (CICR) from the inositol triphosphate and ryanodine receptor located in the endoplasmic reticulum (ER) [4, 5]. To investigate the role of G protein coupling in α7 nAChR modulation by T30, we compared the efficacy of T30 in three cell-based parameters in the α7_345–348 A_ mutant (α7_Gm_) as compared to their overexpressed (α7+) wild-type. The three functional assays performed were T30 induced calcium influx [14, 15], subsequent decrease in cell viability owing to calcium induced excitotoxicity [14, 15, 20], and the compensatory release of AChE from the extant PC12 cells [14, 15, 20]; that have been previously established as a method of investigating T14/T30 induced effects in undifferentiated PC12 cells [14, 15, 20, 21].

Three preparations are compared in this study. First, undifferentiated PC12 cells vs. nerve growth factor (NGF)-differentiated PC12 cells were compared. Second, PC12 cells transfected with an overexpression of the T30 target α7 nAChR were compared to their wild type counterparts. Finally, PC12 cells transfected with a α7_345–348 A_ mutant nAChR vs. PC12 cells transfected with an overexpression of the T30 target α7 nAChR were compared. Ex vivo rat brain slices were subsequently used to investigate whether T30 can regulate changes to the α7 nAChR at the mRNA level in a physiological neuronal population, as has been demonstrated previously *in-vitro* [18]. We also investigated brain samples from AD patients to evaluate disease progression in context of α7 nAChR-T14 relationship. Accordingly, we investigated the protein levels of α7 nAChR and T14 across different Braak stages, and the potential correlation between the two.

## Methods

### PC12 Cell Culture

Wild type PC12 cells were plated in 100 mm dishes coated with type IV collagen from human placenta and cultured in Dulbecco’s Modified Eagle’s Medium (DMEM), supplemented with serum and antibiotics as previously described [21]. For differentiation into neuronal-like cells, nerve growth factor (NGF) was added to the PC12 cells in low serum conditions as previously described [21]. The medium was changed every 2–3 days and the cells were maintained in a humidified incubator at 37 °C with 5% CO_2_. For passaging, the cells scrapped from the dish using a cell scrapper and passed through a needle and syringe, with a fraction of them being plated onto a new dish. For differentiation, differentiation medium was used to culture the cells. PC12 cells were used between passage number 12–20. T30 was synthesized by Genosphere (France) and was used at different concentrations for different experimental paradigms.

### DNA Transfection

Cells were grown to 70–80% confluence in a collagen-coated 35 mm dish. On the day of transfection, the cells were transfected using Lipofectamine 2000 according to the manufacturer’s protocol (Thermo Fisher) using 2 µg of DNA in plain DMEM (serum and antibiotics free). 6 h after the transfection, the medium was replaced by full serum and antibiotics medium, and the cells were plated in a 96-well plate. 48 or 72 h after the transfection, the assay was conducted on the cells or cells were harvested for RNA extraction to confirm and quantify the increase in α7 nAChR mRNA following transfection. Plasmids used in this study have been previously characterized: human α7 nAChR and human α7_345–348 A_ [4–6]. Plasmid DNA was initially obtained from Kabbani lab and replicated using One Shot TOP10 Chemically Competent E. coli bacteria (Life Technologies) and isolated using a Plasmid Mini kit (Qiagen). Plasmids were verified by Sanger sequencing and agarose gel electrophoresis. GFP transfected cells were used as a negative control (Promega, Monster Green® Fluorescent Protein phMGFP Vector, E6421).

### Calcium Fluorometry

Calcium fluorometry was conducted as previously described [14]. Undifferentiated PC12 cells were plated in a 96 well microplate in full medium 2 days before the experiment was conducted. On the day of the experiment, fluo-8 assay buffer was prepared according to manufacturer’s protocol (Abcam, Cambridge, UK, 112,128), and 0.1% of fluo-8 dye was added to the solution. Full medium was removed from the 96 well microplate and replaced by 70 µL of the fluo-8 assay buffer with the peptides or vehicle control, following which the microplate was incubated at 37 °C for 30 min, followed by incubation at RT for 1.5 h in total darkness. For each well, a basal reading was determined, followed by an acetylcholine injection (at 53.3 µM) using a fluorescent plate reader (Fluostar, Optima, BMG Labtech, Ortenberg, Germany). The Ex/Em fluorescence intensity was measured at 490/525 nm. For data-analysis, the basal reading was subtracted from the max reading following the acetylcholine injection, and each value was represented as a percentage of vehicle control.

### Cell Viability Assay

Cell viability was determined using the cell counting kit – 8 (CCK-8; Sigma Aldrich, Merck, kGaA, Darmstadt, Germany, 96,992) as previously described [14]. Briefly, undifferentiated PC12 cells were plated in a 96 well microplate 1 day before the assay in full medium. On the day of the assay, the cells were treated with appropriate concentrations of different peptides/drugs or vehicle control in full medium. After a 3 h incubation with the peptides, the CCK-8 dye was added to each well (10 v/v), followed by an incubation of 60 min at 37 °C. The absorbance reading was then read using a CLARIOStar Plus Plate Reader (BMG LabTech, Aylesbury, UK) at 450 nm. For data analysis, each value was represented as a percentage of the vehicle control.

### AChE Activity Assay

AChE activity was measured using the AChE assay kit (Merck, kGaA, Darmstadt, Germany, MAK119), as previously described [14]. Briefly, PC12 cells were plated in a 96-well microplate 2–3 days before the assay was conducted in full medium. On the day of the assay, the medium was replaced with peptides/drugs or vehicle control dissolved at appropriate concentrations in HEPES (10 mM) supplemented Hank’s Balanced Salt’s Solution (HBSS). After 3.5 h incubation with the peptides in HBSS, the supernatant was removed from the wells and added to another 96-well microplate, to which assay buffer (with assay reagent) was added. The plate was then incubated at RT for 2 min, following which its initial absorbance was read using a CLARIOStar Plus Plate Reader (BMG LabTech, Aylesbury, UK) at 405 nm, and a final absorbance after 8 min. For data analysis, the initial absorbance for each well was subtracted from the final absorbance, and each value was represented as a percentage of the vehicle control.

### Ex Vivo Brain Slices

The rat (P21 male Wistar) brain slicing, and the incubation were performed as described previously [21]. All procedures were performed in accordance with the guidelines provided by the UK Home Office regulations (Schedule 1) and conducted in compliance with the requirements of the UK animals (Scientific Procedures) Act 1986. Briefly, the brain was sliced using a vibratome and 400 μm thick hemisections were collected containing the substantia nigra (SN) region of the rat within the following stereotaxic coordinates: −4.80 to − 6.20 mm from Bregma. Each slice was then divided at the midline to provide two complementary halves. The hemisections were incubated, for 5 h, either with “recording” artificial cerebrospinal fluid (aCSF) alone or treated with T30 (2µM). The composition of the slicing and recording aCSF were used as previously described [21].

### Real-Time Quantitative Polymerase Chain Reaction (RT- qPCR)

Total RNA was extracted from brain tissue using the RNeasy Lipid Tissue Mini Kit, and from cells using RNeasy Plus Mini kit (Qiagen, Manchester, UK) as previously described [21]. cDNA synthesis was conducted using the qScript cDNA Synthesis Kit (95,047, QuantaBio, Beverly MA, USA) as previously described using 2000 ng of RNA from brain tissue and 250 ng of RNA from cells [21]. RT-qPCR was conducted on a Q thermal cycler (Quantabio) as previously described [21] using primers listed in Table 1 and the PerfeCTa SYBR Green FastMix (Quantabio, #95074-012). Relative mRNA levels were quantified using the Q software (Quantabio) with the standard curve method, and target gene expression was normalized to *Gapdh* expression for rat brain tissue, and *Rpl19* and *Beta actin* for cells.

**Table 1:** Primers used for RT-qPCR

Gene	Forward primer (5’-3’)	Reverse primer (5’-3’)	Product size (bp)	Species
*Chrna7*	TCACTGGACCTGCAAATGC	TGACATCTGGGTATGGCTC	133	Rat
*Gapdh*	GGGCTCTCTGCTCCTCCCTGT	CAGGCGTCCGATACGGCCAAA	119	Rat
*CHRNA7*	ACCACTCACCGACTTCC	CATCTGGGAAACGAACAGTCTI	167	Rat and human
*Rpl19*	ATCGCCAATGCCAACTCT	GAGAATCCGCTTGTITTTGAA	321	Rat
*Beta actin*	CCACACGCCACCAGTTCG	TACAGCCCGGGGAGCATCGT	112	Rat

### Western Blotting

Total protein was extracted from human brain using PBS, supplemented with 1x protease (cOmplete™ ULTRA Tablets, Mini, EDTA-free, EASYpack Protease Inhibitor Cocktail, Roche) and phosphatase (PhosSTOP, Roche) inhibitor cocktails by mechanical disruption using KIMBLE Dounce tissue grinder set (Sigma, D9063). The homogenate was then centrifuged at 13,000 RPM at 4 °C for 40 min. The supernatant was collected, and protein was quantified using the Pierce 660 nm Protein Assay (Thermo Scientific). Protein separation by electrophoresis and transfer to PVDF membrane was conducted as previously described [21]. After the transfer, ponceau S stain (Abcam, Cambridge, UK) was used to determine the total protein levels. The membranes were then blocked in milk and incubated in primary antibodies overnight as previously described [21]. The primary antibodies used were against T14 (1:1000, custom made by Genosphere), or anti- α7 nAChR (1:1000, Abcam, ab216485). After primary antibody incubation overnight, the membranes were washed as previously described [21] and incubated with secondary goat-anti-rabbit IgG H + L Horseradish peroxidase (HRP) conjugated antibody (1:10,000, Thermo Fischer Scientific, G21234). Following secondary antibody incubation, the membranes were washed again and an enhanced chemiluminescence based detection kit (BioRad, 1,705,061) was used to visualize the chemiluminescent signal using a CCD Camera (G-Box, Syngene, Cambridge, UK) gel system as previously described [21]. Band intensities were quantified using ImageJ and target proteins were normalized to total protein levels.

### Statistical Analyses

At least three independent experiments were performed for all PC12 assays. Data for the three cell-based parameters were expressed as percentage of vehicle control cells. Statistical analysis of the log transformed data was performed with GraphPAD Prism 9 software using 2-way Analysis of variance (ANOVA) followed by Šídák’s multiple comparisons post-hoc tests, or one-way ANOVA followed by Tukey’s multiple comparisons post-hoc tests. Ex vivo rat data were analyzed using Wilcoxon matched-pairs signed-ranks test, comparing left vs. right hemisphere of the same brain section to compare treated halves vs. control halves. *Post-mortem* human brain data were analyzed using Pearson’s correlation. Statistical significance was considered at *P* value < 0.05 for all tests. All PC12 results in the graphs are presented as mean ± SEM.

## Results

### NGF-Differentiated PC12 Cells are Less Sensitive to T30

**Fig. 1:**
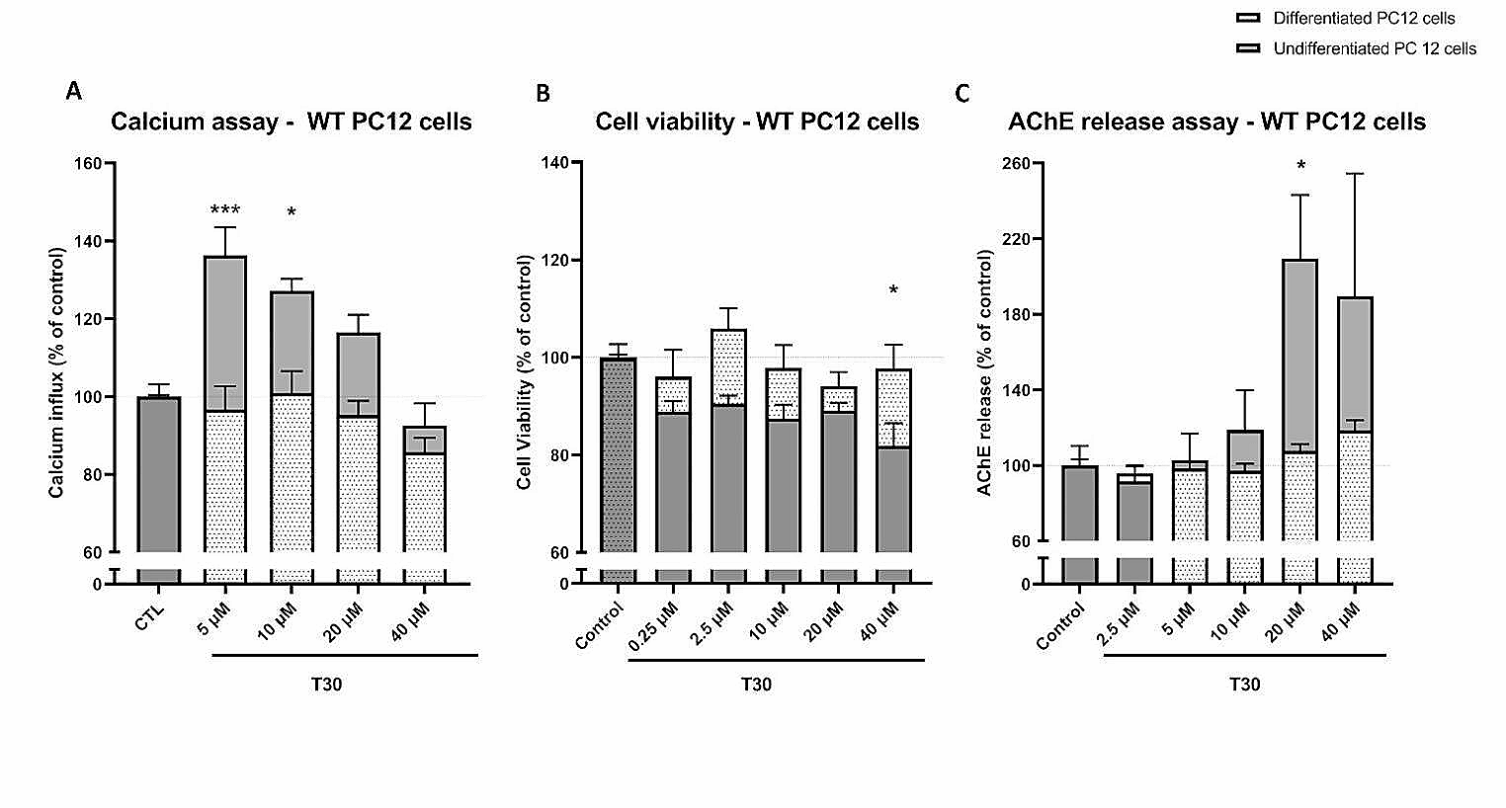
Differentiated PC12 cells are less sensitive to T30. (**A**) Calcium influx, (**B**) cell viability and (**C**) AChE release in differentiated and undifferentiated PC12 cells following treatment with different T30 concentrations. For each concentration, 3–11 replicates of PC12 cells were cultivated and used to assess the drug response in each assay (mean ± SEM). These three parameters are expressed as % of vehicle control treated cells. Comparisons between the differentiated vs. undifferentiated at multiple-concentrations (*N* = 3–11) were performed by two-way analysis of variance (ANOVA, factor “differentiation”, factor “concentration” and their interaction) followed by šídák’s multiple comparisons post-hoc tests to determine the significance of the differentiation effect for each concentration, in comparison to undifferentiated cells. *: *P* < 0.05; ***: *P* < 0.001

We wished to confirm whether NGF-induced differentiation of PC12 cells impacted their response to T30. In the wild type (WT) PC12 cells, undifferentiated cells showed a significant increase in calcium influx at T30 concentrations of 5 and 10 µM, and a gradual decrease from the concentrations of 10 µM to 40 µM (Fig. 1A; two-way ANOVA, factor “T30 concentration”: *P* = 0.0003, F_3,28_ = 8.623; factor “differentiation”: *P* < 0.0001, F_1,28_ = 30.32; interaction “concentration x differentiation”: *P* = 0.1339, F_3,28_ = 2.02). 5 µM T30 induced a calcium increase of 136% in the undifferentiated cells, whereas T30 did not increase the calcium influx significantly above control in the differentiated cells at any concentration tested. For the cell viability parameter, undifferentiated cells showed a significant decrease of cell viability in response to T30 (down to 81%, at 40 µM) compared to the differentiated PC12 cells (Fig. 1B; two-way ANOVA, factor “T30 concentration”: *P* = 0.4336, F_4,41_= 0.9715; factor “differentiation”: *P* = 0.0004, F_1,41_ = 14.85; interaction “concentration x differentiation”: *P* = 0.6574, F_4.41_= 0.6105). For the T30-induced AChE release, only the undifferentiated cells showed a concentration-dependent increase of AChE up to 209% of control at 20 µM, in contrast to the differentiated PC12 cells (Fig. 1C; two-way ANOVA, factor “T30 concentration”: *P* = 0.0046, F_4,30_ = 4.704; factor “differentiation”: *P* < 0.0001, F_1,30_ = 6.239; interaction “concentration x differentiation”: *P* = 0.117, F_4,30_ = 2.019).

### PC12 Cells with α7 nAChR Overexpressed Exhibit a Larger Calcium Response to High T30 Concentration

**Fig. 2:**
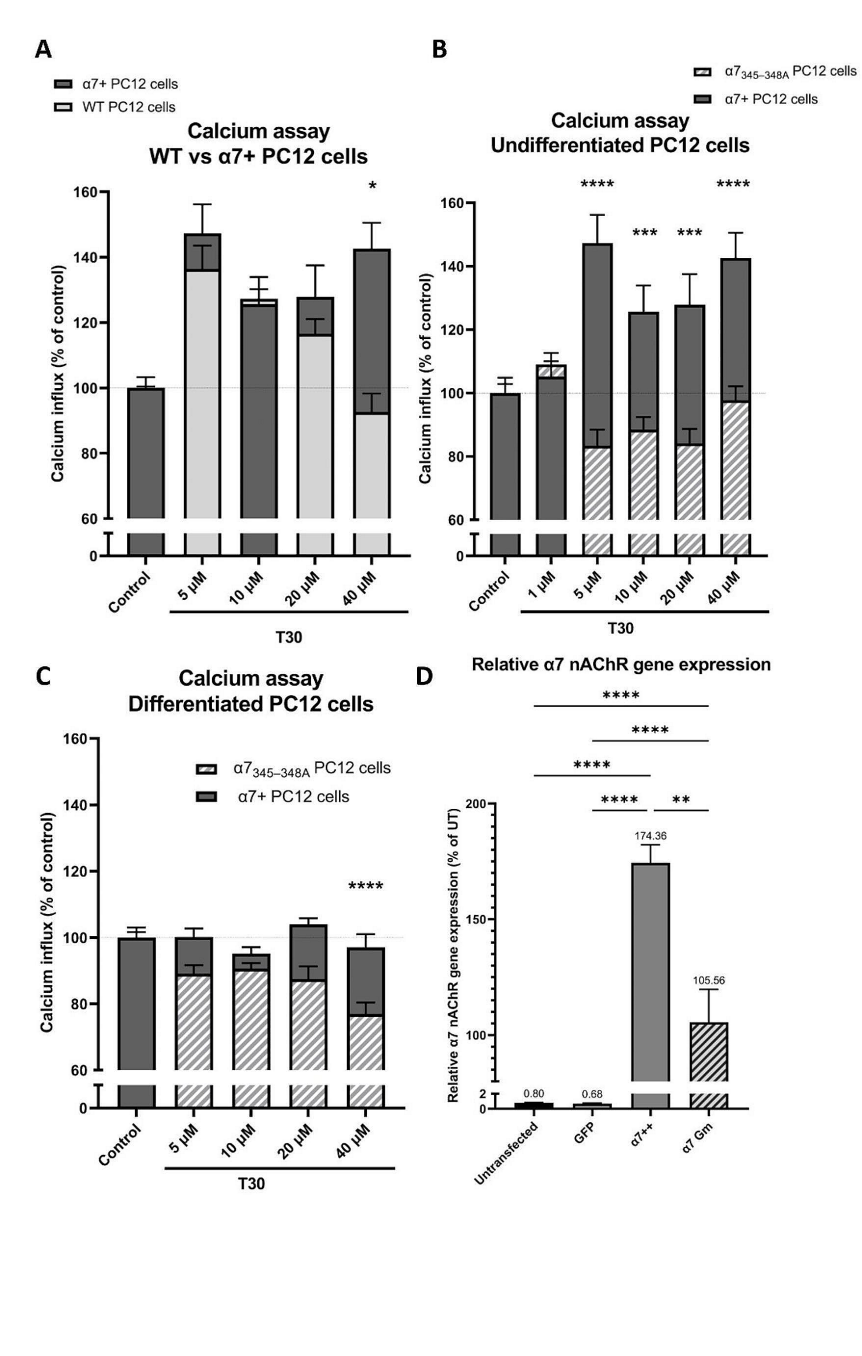
The mutation in the GPBC cluster of α7 nAChR affects the T30-dependant calcium influx. PC12 cells were transfected with either wild type human α7 nAChR plasmid (α7 + cells) or a plasmid with a mutation in the GPBC of the α7 nAChR (α7_345–348 A_) which acts as a dominant negative. Different T30 concentrations were used on undifferentiated (**A**, **B**), and on differentiated PC12 cells (**C**): for each concentration, 3–24 replicates of PC12 cells were cultivated and used to assess the drug response in each assay (mean ± SEM). The calcium parameters are expressed as % of vehicle control cells. Comparisons between the α7 + vs. WT, and the α7 + vs. α7_345–348 A_ at multiple-concentrations (*N* = 3–24) were performed by two-way analysis of variance (ANOVA, factor “mutation”, factor “concentration” and its interaction) followed by šídák’s multiple comparisons post-hoc tests to determine the significance of the mutation in comparison to α7 + cells, per concentration. (**D**) Average gene expression of α7 nAChR was determined by RT-qPCR (*N* = 3 per group). *: *P* < 0.05; **: *P* < 0.001; ***: *P* < 0.001; ****: *P* < 0.0001

Undifferentiated PC12 cells were transfected with the wild type human α7 nAChR plasmid (α7 + cells) to compare T30 induced calcium influx to undifferentiated un-transfected wild type (WT) PC12 cells. In the undifferentiated cells, when comparing the calcium profile of the overexpressed α7 nAChR (α7+) cells with the un-transfected WT (Fig. 2A), the α7 + cells showed a higher calcium response at the highest concentration of 40µM (143% vs. 93%). However, at the lower concentrations (< 40µM), T30 showed a comparable profile between the α7 + and WT cells (overall two-way ANOVA, factor “T30 concentration”: *P* = 0.3145, F_3,77_= 1.203; factor “overexpression”: *P* = 0.168, F_1,77_ = 1.937; interaction “concentration x α7+”: *P* = 0.1722, F_3,77_ = 1.709).

### Mutation in the GPBC of α7 nAChR Affects T30-Induced Calcium Influx in a Concentration-Dependent Manner

We wished to establish the role of the GPBC in T30 induced calcium influx. PC12 cells were transfected with either wild type human α7 nAChR plasmid (α7 + cells) or a plasmid with a mutation in the GPBC of the α7 nAChR (α7_345–348 A_) which acts as a dominant negative, suppressing the endogenous expression of α7 nAChR [4–6]. RT-qPCR analysis was performed using primers which recognize both human and rat α7 nAChR mRNA, to detect both endogenous mRNA expression and transfected mRNA expression. This demonstrated successful upregulation following transfection with the α7 nAChR plasmid (Fig. 2D; one-way ANOVA, factor “Plasmid”: *P* < 0.0001, F_3,8_ = 2.116; followed by Tukey’s multiple comparisons post-hoc tests). For the transfected undifferentiated PC12 cells, the overexpressed α7 + showed a significant calcium increase up to 147% for the T30 concentrations of 5–40 µM compared to control treated. Whereas the mutated α7_Gm_ did not increase calcium above control level in response to T30 (Fig. 2B; two-way ANOVA, factor “T30 concentration”: *P* = 0.1097, F_4,181_ = 1.916; factor “mutation”: *P* < 0.0001, F_1,181_ = 76.04; interaction “concentration x mutation”: *P* < 0.0001, F_3,28_ = 9.867). For the transfected differentiated PC12 cells, the mutated α7_345–348 A_ showed a significant calcium decrease (down to 76%) in response to T30 compared to the α7+, at the concentration of 40 µM (Fig. 2C; two-way ANOVA, factor “T30 concentration”: *P* = 0.0176, F_3,71_ = 3.598; factor “mutation”: *P* < 0.0001, F_1, 71_ = 19.18; interaction “concentration x mutation”: *P* = 0.1671, F_3,71_ = 1.738).

### Mutation in the GPBC of α7 nAChR Blocked the Effects of T30 on Cell Viability and AChE Release

**Fig. 3:**
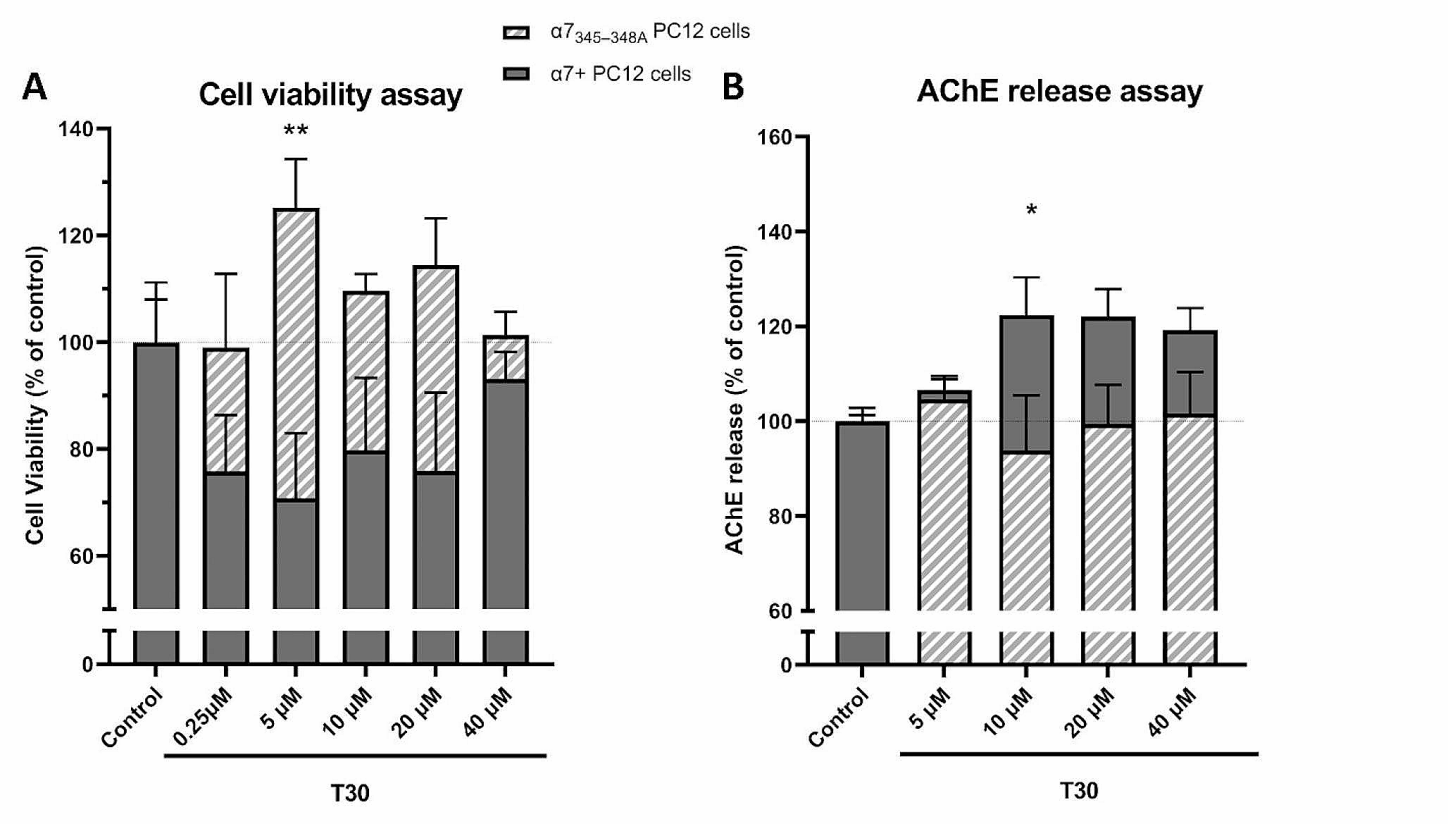
The mutation in the GPBC cluster of the α7 nAChR affects the T30-dependant metabotropic responses. The effect of different T30 concentrations on cell viability (**A**) and AChE release (**B**) in PC12 cells transfected with either wild type human α7 nAChR plasmid (α7 + cells) or a plasmid with a mutation in the GPBC of the α7 nAChR (α7_345–348 A_). For each concentration, 3–19 replicates of PC12 cells were cultivated and used to assess the drug response in each assay (mean ± SEM). The cell viability and AChE parameters are expressed as % of vehicle control-treated cells. Comparisons between the α7 + vs. α7_345–348 A_ at multiple-concentrations (*N* = 3–19) were performed by two-way analysis of variance (ANOVA, factor “mutation”, factor “concentration” and its interaction) followed by šídák’s multiple comparisons post-hoc tests to determine the significance of the mutation in comparison to α7 + cells, per concentration. *: *P* < 0.05; **: *P* < 0.01

We wished to establish the role of the GPBC on T30 induced excitotoxicity, i.e., a decrease in cell viability, and the compensatory increase in AChE release by extant PC12 cells. PC12 cells were transfected with either wild type human α7 nAChR plasmid (α7 + cells) or a plasmid with a mutation in the GPBC of the α7 nAChR (α7_345–348 A_) which acts as a dominant negative, suppressing the endogenous expression of α7 nAChR [4–6]. For the cell viability parameter, the overexpressed α7 + cells exhibited a significant reduction in cell number for the T30 concentrations of 0.25 µM − 20 µM, compared to vehicle control. Whereas the mutated α7_345–348 A_ did not reduce cell viability at any T30 concentration tested (Fig. 3A; two-way ANOVA, factor “T30 concentration”: *P* = 0.7153, F_4,54_ = 0.5284; factor “mutation”: *P* < 0.0001, F_1,54_ = 24.1; interaction “concentration x mutation”: *P* = 0.0989, F_4,54_ = 2.06), suggesting that the mutation attenuated T30-induced cell loss at all concentrations. For the T30-induced AChE release, the overexpressed α7 + showed a significant increase in AChE up to 122% for the concentrations ranging from 1 to 40 µM, whereas the mutated α7_345–348 A_ abolished the T30-induced AChE at all concentrations (Fig. 3.B; two-way ANOVA, factor “T30 concentration”: *P* = 0.5639, F_5,66_ = 0.7854; factor “mutation”: *P* = 0.0001, F_1,66_ = 16.79; interaction “concentration x mutation”: *P* = 0.1724, F_5,66_ = 1.6).

### T30 Drives an Increase in α7 nAChR mRNA in ex Vivo Brain Slices

**Fig. 4:**
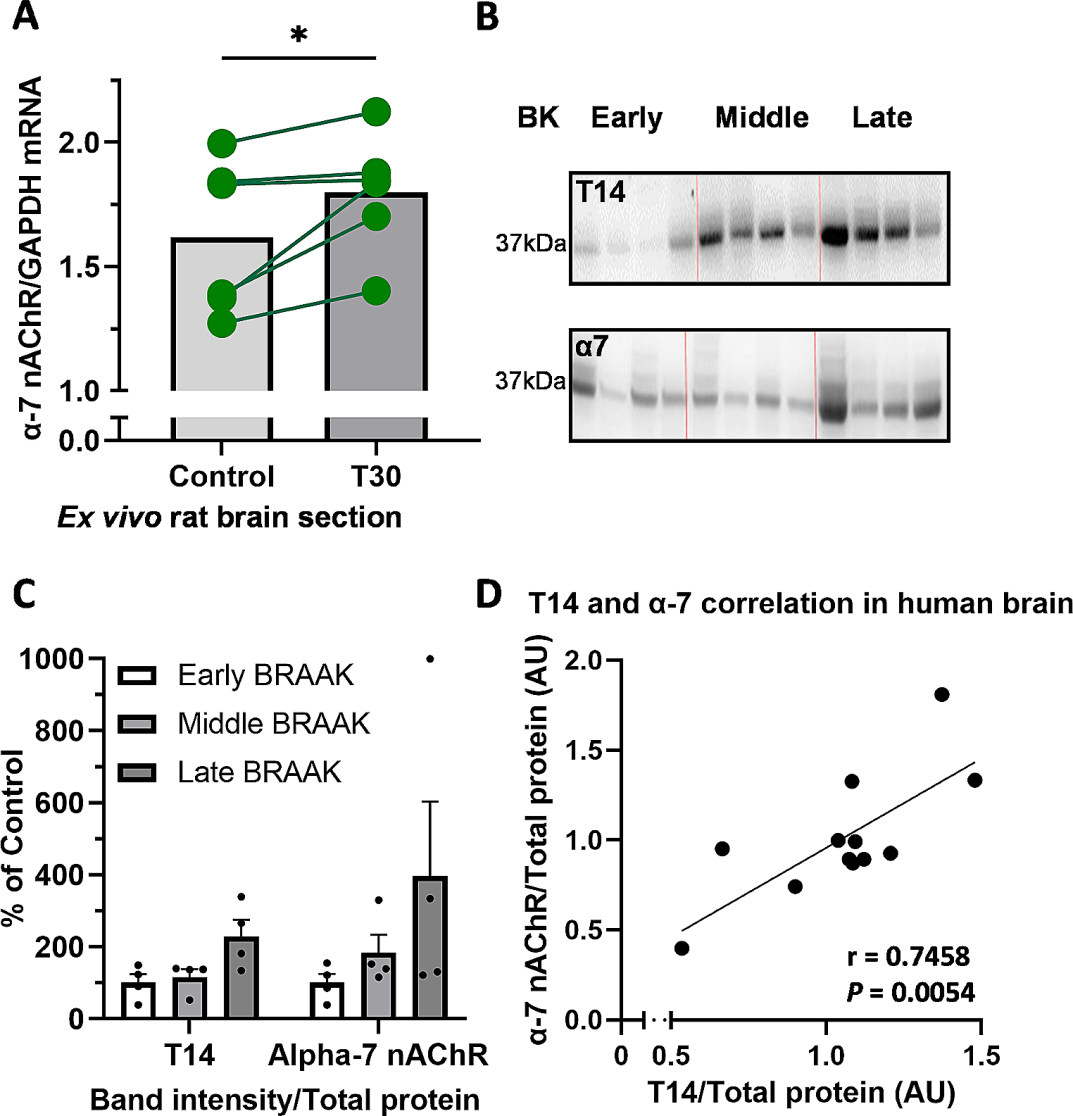
α7 nAChR protein levels correlate with T14 in different physiological preparations. **A**) T30 upregulates α7 nAChR mRNA expression in rat ex vivo brain slices containing substantia nigra. Comparison between the T30 (2 µM) and vehicle control treatments (*N* = 6) was performed by a Wilcoxon matched-pairs signed rank test, to determine the significance of the T30 treatment: * *P* < 0.05. **B**) Chemiluminescent signal detected using western blot for α7 nAChR (37 kDa) and T14 (37 kDa) in human hippocampus. **C**) Bar plot showing the relative values of T14 and α7 nAChR protein, normalized to the total protein stain ponceau in post-mortem human brain hippocampus from AD patients (early Braak: Braak stages 0, 1, 2; middle Braak: Braak stages 3, 4; late Braak: Braak stages 5, 6). Mean ± SEM; % of control; *n* = 4. **D**) T14 levels correlate with α7 nAChR protein levels (Pearson’s correlation: *r* = 0.7458, *P* = 0.0054) detected in human post-mortem human brain hippocampus. Both T14 and α7 were normalized to the total protein, as arbitrary units (AU)

We have previously shown that T30 drives an increase in α7 nAChR at the protein level in the basal forebrain of the rat brain ex vivo [17]. We aimed to determine if T30 application increased the expression of α7 nAChR at the mRNA level in ex vivo rat brain slices containing the substantia nigra. Half of the rat brain slice was treated with aCSF alone, and the other half of the brain slice was treated with T30 added in aCSF (at 2 µM). T30 induced a significant increase in expression of α7 nAChR mRNA (Fig. 4A; Wilcoxon matched pairs signed rank test, two tailed, *P* = 0.0313; Pairing effectiveness: rs = 0.9429, *P* = 0.0083).

### Correlation of α7 nAChR and T14 in Human Brain Hippocampus

To further investigate the relationship between T14 and its target receptor in a clinically relevant experimental paradigm, we used western blotting to determine the levels of T14 and α7 nAChR in post-mortem human brain from AD patients. In the post-mortem human brain hippocampus from AD patients, both T14 and α7 nAChR exhibited an increase at the protein level as the disease progressed with increasing Braak stages (Fig. 4B and C). Furthermore, levels of T14 and α7 nAChR were significantly correlated with each other (Fig. 4D: Pearson’s correlation, *r* = 0.7458, *P* = 0.0054, *N* = 12).

## Discussion

### The Link between T14 and α7 nAChR

Here, we investigated the role of the α7 nAChR induced metabotropic signaling in T14 induced process in PC12 cells. Previous work from a wide range of preparations has suggested a close and selective link between the α7 nAChR and T14. In oocytes transfected with α7 nAChR, T14 enhanced calcium influx in the presence of a primary ligand; however, this response was not observed in oocytes transfected with the closely related α4/β2 nAChR [8]. T14/T30 also drove an increase in the expression of α7 nAChR mRNA and increased the translocation of the α7 nAChR to the membrane from the cytosol in human GH4-Hα7 cells [18]. In the ex vivo rat brain, T30 application led to an increase in the expression of α7 nAChR at the protein level [17]. Moreover, when detected via voltage sensitive dye imaging (VSDI), T30 had a site-selective bioactivity in the basal forebrain and substantia nigra, which are rich in α7 nAChR; in contrast the peptide was ineffective in the striatum, an area where nicotinic receptors are in general abundant, but where the α7 nAChR specifically is absent [22]. Furthermore, the effect of T30 on neurite growth in organotypic hippocampal cultures from rat brain was blocked by the traditional α7 nAChR antagonist, α-bungarotoxin [8]. In summary, there is already persuasive evidence to support the suggestion that T14 acts via the α7 nAChR, however little is known regarding the G protein coupling mediated subsequent intracellular events.

### Rationale for Experimental Protocol

In this study, we investigated the intracellular effects triggered by activation of the α7 nAChR by the T30 peptide, purportedly pivotal in AD [13]. The three *in-vitro* parameters established for demonstrating the efficacy of T30 were: first, the ionotropic mechanism, as seen by enhanced calcium influx in response to an agonist, such as acetylcholine [8, 15]; second, subsequent reduction in cell viability due to the ensuing excitotoxicity from calcium excess [14, 15, 20]; and third, an increased compensatory release in AChE from neighboring surviving cells [14, 15]. The two latter mechanisms may be related to the metabotropic mechanism of the α7 nAChR or may be a result of raised levels of calcium, or both.

PC12 cells, derived from rat adrenal medulla, offer one of the most valuable mammalian cell models to study the α7 nAChR [14], and have been widely used to study nervous system disorders (including AD and Parkinson’s Disease) and the mechanisms of drug action [23, 24]. PC12 cells exhibit a plethora of neuronal features, including the ability to differentiate into a sympathetic neuron-like phenotype [24]. They can synthetize and store neurotransmitters, including acetylcholine, deficiency of which is implicated in AD [23, 25–27]. In addition, PC12 cells are amenable to genetic manipulations, such as transfection or viral transduction, which allow the introduction of specific genetic modifications to overexpress/knockdown genes of interest [5]. This feature of PC12 cells enabled the investigation of how the specific genetic alteration of α7 nAChR contributes to the bioactivity of T14 and hence the possible pathogenesis of AD.

To complement the *in-vitro* work performed in PC12 cells to investigate the role of α7 nAChR in T14 induced process, we used additional, more physiological experimental setups: ex vivo rat brain slices and the *post-mortem* human brain hippocampus. Ex vivo rat brain slices have been previously established as a useful preparation to measure biochemical changes in response to T30 in a physiologically relevant neuronal population [17, 28]; allowing site-specific investigation of bioactive agents whilst the symmetry of the coronal section about a midline allows for an ideal control where only one side is treated and compared with its counterpart. Slices containing substantia nigra were used as T30 is bioactive in this region [22].

In the human brain study, to investigate the relationship between T14 and α7 nAChR, the hippocampus was evaluated, given its importance in memory function, and dysfunction in AD [29]. Western blotting was utilized to investigate the relative levels of these proteins with a custom-made polyclonal T14 antibody previously validated extensively to only recognize the free T14 sequence [15, 30]. We have demonstrated that T14 levels increase in the AD hippocampus [13], however, we have not yet explored the link between α7 nAChR and upregulation of T14 in the AD brain. The use of human hippocampus samples here allowed us to track the expression of these proteins as a function of the degenerative process.

### Effects of PC12 Differentiation on T30 Sensitivity

PC12 cells have two distinct phenotypes: undifferentiated and differentiated [23]. Undifferentiated PC12 cells synthesize catecholamines [23]. In the presence of NGF, PC12 cells differentiate into sympathetic nerve-like cells, which are similar to neurons in terms of morphology, physiological and biochemical functions [23, 26]. Differentiated PC12 cells produce more acetylcholine (ACh) at the expense of norepinephrine [31]. Higher levels of basal ACh in NGF-differentiated PC12 cells may therefore render them less sensitive to T30 due to the possibility that more α7 nAChRs could be apparent in the desensitized state [32]. Indeed, the data in this current study support this, where NGF-differentiated PC12 cells did not respond to a PAM of the α7 nAChR, i.e., T30. In addition, many studies have shown that NGF contributes to the survival and regeneration of neurons during ageing and neurodegenerative conditions such as AD [33–35]. Changes in neurotrophic signaling pathways are involved in the ageing process and contribute to reduced cholinergic transmission and cognitive decline as observed in AD [36–38]. In fact, the potential anti-inflammatory property of NGF in the context of AD could be protective against T30-induced toxicity in NGF-differentiated PC12 cells [35]. The data presented here are consistent with NGF induced neuroprotective effects, as NGF-differentiated PC12 cells were less susceptible to T30 induced excitotoxity.

The neurons that degenerate primarily at the onset of AD are within the interconnecting subcortical cell groups referred to by Woolf (1996) as ‘Global neurons’ [39], located within the brain stem and the midbrain [40]. From the embryonic stage as early as 4 weeks gestation, these potentially vulnerable nuclei can be distinguished from the other neurons, by their provenance i.e., the basal plate rather than the alar plate [39]. A defining feature of the ‘global neurons’ is that they retain their developmental potential into adulthood, and an increased sensitivity to trophic agents, which is not seen in their alar-plate derived counterparts [19, 39]. This persistent capacity for growth could explain why, in the mature brain, these cells will be the first to be affected by the gradual neurodegeneration [40]. It is here, in the basal plate derived cells where there are high levels of AChE irrespective of its classical substrate ACh [41]. AChE has long been proposed to have non-enzymatic functions, especially in the ‘Global neurons’, where it is co-expressed strongly with the α7 nAChR [16]. NGF-differentiated PC12 cells could therefore be considered a model of mature neurons [24], and indeed exhibited lesser sensitivity to T30 here. In contrast, undifferentiated PC12 cells, which retain their developmental potential, i.e., ability to proliferate and differentiate [24], were more sensitive to T30 effects; this observation is consistent with the hypothesis that T14/T30 initiates the neurodegenerative process in the ‘Global neurons’, separately derived from embryonic basal plate retaining their developmental potential into adulthood.

### Effect of α7 nAChR Overexpression and G Protein Mutation in PC12 Cells

Overexpression of α7 nAChRs in PC12 cells has been previously shown to result in an increased or longer lasting calcium influx, which can trigger various intracellular signaling pathways [3, 4, 6]. In addition, overexpression of α7 nAChRs in PC12 cells may also affect the release of various neurotransmitters, including ACh, potentially altering neuronal communication and synaptic function [42]. In our study, when the α7 nAChRs were overexpressed in the PC12 cells, the calcium influx in T30-treated cells was higher than the WT (142% vs. 92%)at the ighest concentration of T30 (40µM). At the highest concentration in WT PC12 cells, T30 inhibits calcium influx, as demonstrated previously in α7 nAChR transfected oocytes and PC12 cells [8, 14], most probably due to channel inactivation and/or channel desensitization [43]. Following an increase in the number of α7 nAChR, T30 proved to be more efficacious at 40 µM when compared with the WT (un-transfected) PC12 cells. This effect could be due to an equilibrium state existing between the amount of T30 and the α7 nAChR, which reaches a ceiling effect at 5 µM in WT PC12 cells with endogenous α7 nAChR expression, and then at higher concentrations is desensitized.

However, by increasing the availability of T30 target, i.e., the α7 nAChR, higher concentrations of T30 do not desensitize the receptor, suggesting that T30 acts via the α7 nAChR. It is interesting to note that despite increasing the availability of the α7 nAChR, the magnitude of T30-induced calcium influx across all concentrations was not significantly much higher than WT PC12 cells with endogenous α7 nAChR expression, hence a ceiling effect may be in operation. This effect has also been observed by other studies investigating PNU120596 induced calcium influx in PC12 cells with endogenous α7 nAChR expression compared to the cells with α7 nAChR over-expressed [5]. Further experiments corroborate the evidence that T30 is acting upon α7 nAChR, where it is potentially toxic at high concentration due to a calcium excess [44, 45]. Indeed, T30 excitotoxicity is confirmed by the effects on cell viability, that measures a more downstream response, with the highest cell reduction down to 70% (obtained at 5 µM) in the α7+, as compared to only 87.5–90% (at 2.5–10 µM) in the WT PC12 cells.

In this current study, the role of T30 on the α7 metabotropic signaling responses has also been assessed. We used a well characterized mutation that induces the loss of G protein binding in α7_345–348 A_ [5]. By acting as a dominant negative, this mutation reduces calcium signaling by PNU120596, a potent selective PAM of the α7 nAChR, at the level of three functional readouts- calcium peak, calcium duration and the neurite outgrowth [4–6, 11]. The α7_345–348 A_ transfected PC12 cells were compared to the overexpressed α7+, where the mutant α7_345–348 A_ reduced the T30-induced calcium response. This finding is consistent with the PNU120596-induced calcium increase which was significantly attenuated in the α7_345–348 A_ transfected PC12 cells. The mutant α7_Gm_ also affected the more downstream responses of cell viability and AChE release. Indeed, the subsequent reduction in cell viability due to the ensuing T30 excitotoxicity was fully attenuated by α7_Gm_ and the consequent increase in compensatory AChE release was also abolished in the α7_345–348 A_. This result is consistent with a recently published study, where the T30-induced effects on neurite outgrowth in SH-SY5Y cells were abolished by the expression of the α7_345–348 A_ mutant [11], corroborating the near exclusive relationship reported between T14 and α7 nAChR in PC12 cells our study.

### T30 Induced Positive-Feedback Loop with α7-nAChR

**Fig. 5:**
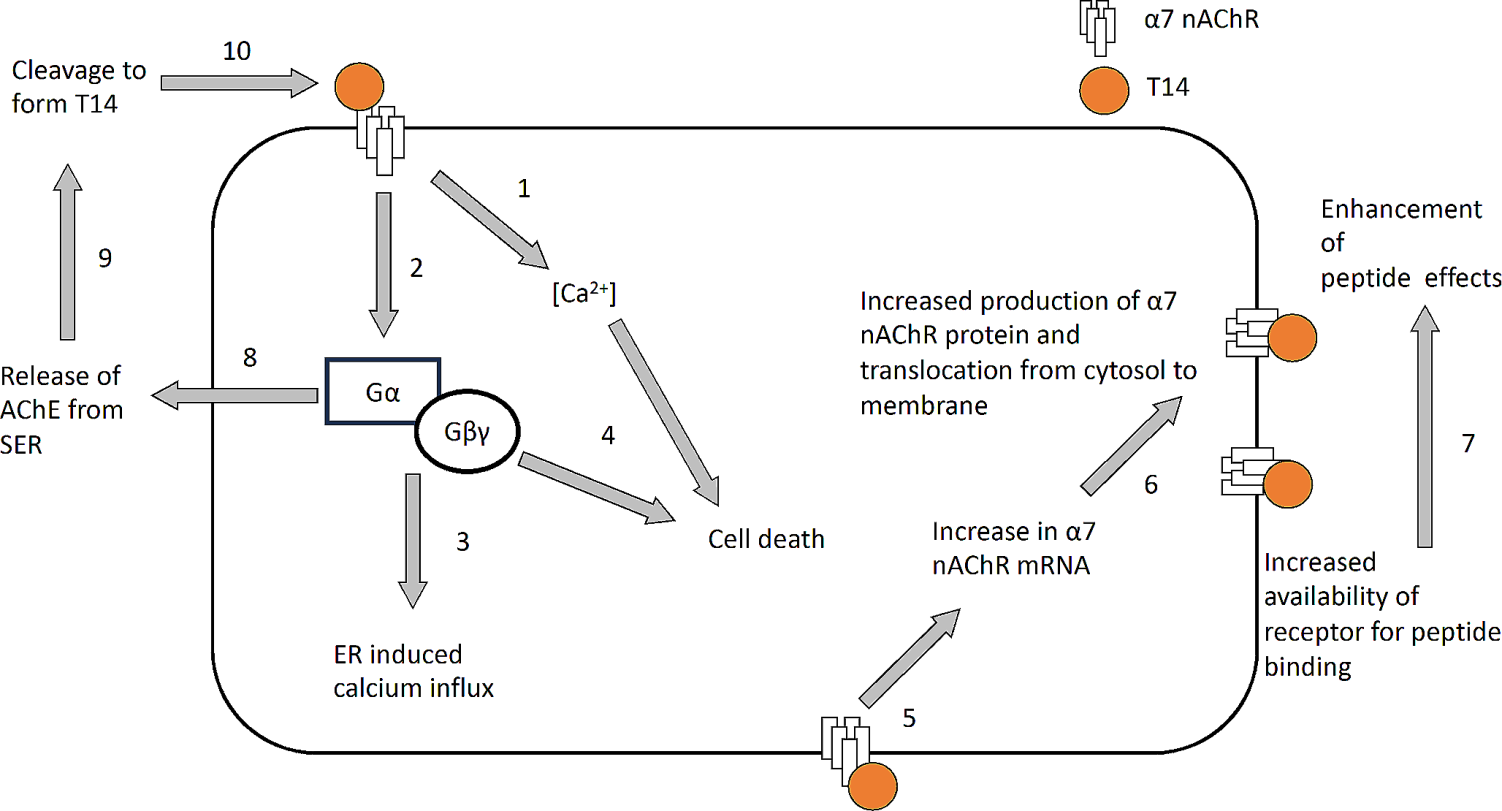
Proposed T14-induced positive feedback loop with the α7 nAChR. T14 leads to α7 nAChR activation, **(1)** which drives calcium influx [8, 14] and **(2)** G protein activation. **(3)** The G protein activation drives enhanced calcium influx via the endoplasmic reticulum (ER) [5], **(4)** leading to cell death, in addition to the calcium induced excitotoxicity [10, 14, 15]. **(5)** T14 also drives an increase in α7 nAChR expression at mRNA level, **(6)** which is translated into high protein levels [11, 18]. Higher protein levels of α7 nAChR are translocated from the cytosol to the membrane [18], **(7)** where the peptide has increased availability of its target receptor, which leads to enhanced effects of the peptide on calcium-induced excitotoxicity. This potentially forms a positive feedback loop, where higher α7 nAChR activation by the peptide leads to increase in α7 nAChR production, leading to increased ensuing excitotoxicity. **(8)** G protein activation also leads to release of AChE from the smooth endoplasmic reticulum (SER) [14, 15, 20], **(9)** which is cleaved to form T14 [12], **(10)** which can further bind with the α7 nAChR ensuing the continuation of the positive feedback loop

In GH4-hα7 cells, Bond et al. [18], previously showed that T14 and T30 peptides induced an overexpression of the α7 nAChRs in both mRNA and protein, as well as at the post-translational level by increasing the translocation of the receptor from cytosol to the membrane. Recently, Graur et al. [11] showed that T30 induced the overexpression of α7-nAChR at the protein level in SH-SY5Y cells; here we demonstrate that α7 nAChR mRNA is increased in the presence of T30 in *ex-vivo* rat brain slices. Taken together, these data suggest that the T14 also increases the production of its own target, i.e., the α7 nAChR. This action could potentially trigger a positive feedback loop, thereby increasing the availability of its target receptor, and ensuring further excitotoxic effects (Fig. 5).

Our findings also provide further insight into the presence of α7 nAChR in AD, which has been inconsistent in previously published literature. At the protein level, α7 nAChR was found to be negatively correlated with amyloid beta (Aβ) in the brain of patients with AD, in the hippocampus, temporal and frontal cortices, with lower α7 nAChR levels detected in AD patients [46]. In contrast, in another study, α7 nAChR protein was significantly higher in the AD group compared to the control group, in peripheral blood leukocytes, which is consistent with our findings [47]. The high co-expression of T14 and α7-nAChR in post-mortem human hippocampus across increasing Braak stages also hints at the existence of a potential positive feedback loop in response to T14-induced activation of the α7-nAChR.

Dysfunction of the α7 nAChR is a key factor in the pathogenesis of AD and its cognitive sequelae [47, 48]. For example, α7 nAChR is involved in regulating amyloid-beta (Aβ) production [1], a hallmark of AD pathology, which has been demonstrated as a downstream effect of T30 application (15, 20, 28). However, drugs targeting the α7 nAChR to treat different neurological conditions have met with only limited clinical success so far, potentially because the key site is already occupied by endogenous T14 [1]. In contrast, the pharmacological targeting of α7 nAChR would be a promising strategy for the T14 blocker, NBP14; since it is an inert and cyclized form of the natural counterpart, T14, which it can displace [13, 28].

## Conclusion

Taken together, these data suggest that T14 acts exclusively via the α7 nAChR in PC12 cells. The enhanced potency with specific over-expression of α7 nAChR and the effect of α7_345–348 A_ mutant on T30-induced effect in all the functional readouts performed support this conclusion. Furthermore, the peptide-induced increase in α7 nAChR mRNA in ex vivo brain slices, and the high correlation between T14 and α7 nAChR in *post-mortem* human brain demonstrate that a positive feedback loop between the α7 nAChR and T14 may be in operation. It is indeed interesting and unusual for a bioactive agent to have a near-exclusive relationship with one receptor, and the data presented in this study would collectively make it hard to account for T14 binding to any other receptor. In summary, this study provides new insights into the cellular mechanisms of T14 and its highly selective target receptor, which may also underlie the basic process of neurodegeneration in AD. By demonstrating the potential for near-exclusivity, this study inspires the development of a relatively rare therapeutic strategy with potentially minimal off-target effects.

## Data Availability

The raw data generated during this study can be requested from the corresponding author upon reasonable request.
